# Annotations

**Published:** 1907-04-27

**Authors:** 


### Annotations.

### Dr. John Carswell.

The sympathy of the entire profession will be freely extended to Dr. J. Carswell in connection with the dastardly outrage of which he has been the victim. It may be remembered that some twelve months ago Dr. Carswell, in association with Dr. Marion Gilchrist, certified as insane a certain Wm. Purves, who, on the certificates granted by the doctors, was for a time confined in an asylum. On his release Purves brought an action for damages against Dr. Carswell and Dr. Gilchrist, and, after a prolonged trial in the Court of Session, a verdict was given entirely exonerating the defendants. As has been the case in other similar instances, it was not possible to recover the costs from the unsuccessful suitor, and as a necessary result each of the medical practitioners concerned had to pay a solicitor's account amounting to several hundreds of pounds. To this penalty for the honest discharge of professional duty undertaken for the protection of the public, Dr. Carswell has now to add the suffering and risk of an attack on his life. Just as he was leaving the hospital on the 16th inst. he was shot by Purves, who had evidently been waiting in the neighbourhood for this purpose. It is only by some strange good fortune that the result was not immediately fatal, as the revolver was used at close quarters, and no fewer than three bullets were lodged in Dr. Carswell's body, one of these just missing the left femoral artery. We are glad to know that so far Dr. Carswell is making favourable progress. Our readers will join us in an expression of sincere sympathy, and in the cordial hope that he will make a rapid and complete recovery. There is much to be said about the state of the law relating to lunacy in Scotland, but this is not, perhaps, the best time to say it. The public must, however, realise that responsibilities undertaken for its safety demand some protection for those on whom the immediate duty falls.

### Mercury in Modern Therapeutics.

A recent discussion on this subject at the Liverpool Medical Institution seems worthy of general attention. The crowd of new remedies, pressed nowadays on the profession by all the resources of ingenious and persistent advertisement, is so great that there is danger of many of the older drugs being largely forgotten. This danger is particularly great in reference to younger practitioners, who have but a scanty stock of personal experience, and some of whom, at all events, manage to acquire the conviction that, apart from a pharmacological justification, no reputed remedy is worth discussion. It is well, therefore, that the experienced clinical physician should make his voice heard, and hence Professor Wm. Carter's paper, in opening the discussion above referred to, is particularly welcome. Apart from the treatment of syphilis, Professor Carter says frankly—and we doubt not with perfect accuracy—mercury is far too much neglected as a therapeutic agent. Even to those who will only heed the results of laboratory experiments his display of the power of calomel to prevent or correct the decomposition of bile must strongly appeal. Such a demonstration, too, is not far removed from what must actually occur when calomel is given by the mouth. For the insolubility of calomel in the stomach permits it to reach the duodenum unchanged, and so to come into immediate contact with the bile and the pancreatic juice, over both of which, as can be shown *in vitro*, it exercises a decided antiseptic action. Doubtless it has a similar effect on many toxins which in various conditions are formed in the bowel, and the absorption of which is responsible for many sensations indicative of ill-health. In chronic cardiac disease, in meningitis, in peritonitis, mercury has a large field of utility, and many a practitioner may find it well-spent time to consult some of the older works on materia medica rather than to occupy himself with the ingenious catalogues of the modern advertising chemist.

### Medical Facts and Figures in Lord Cromer's Report.

It is hardly possible to refer to Lord Cromer's official report without at least a passing word of tribute to the long, efficient, and patriotic services this great public servant has rendered to Egypt and to the cause of humanity. Profound regret will be felt in this country that, as a result of ill-health, these services, but by no means their results, have necessarily been brought to a conclusion. The report in question contains a number of facts and figures which have a special interest to medical readers. Of immediate official work is to be noted the Anti-rabic Institute which, during the eight months over which its operations extended, healed 451 patients, of whom only four died of hydrophobia. There have yet to come the more radical measures which have proved so successful in this country. Sir Ernest Cassel's ophthalmic hospitals, now under Government control, healed during the year some 7,000 new patients, among whom, of course, was a large proportion of the affections of the eyelids and conjunctiva for which Egypt is notorious. Regret is expressed that numerous demands in other directions render it impossible at present to replace the Kasr-el-Ainy Hospital at Cairo by a building more in harmony with modern methods. The Government Medical School appears to have well established its popularity, as the entries numbered 97, though only 23 of the candidates could be admited; the staff has recently been increased, and may now claim to be fairly complete. Three large hospitals are under construction in the Soudan, and a dispensary for sick children, supported by voluntary contributions, has been opened in Cairo. Accommodation for lunatics has also been increased; it appears that many cases of insanity originate in the use of hashish, large quantities of which, in spite of Government prohibition, are imported. Altogether the report has a very wide and varied medical interest.

